# Motile properties of the bi-directional kinesin-5 Cin8 are affected by phosphorylation in its motor domain

**DOI:** 10.1038/srep25597

**Published:** 2016-05-24

**Authors:** Ofer Shapira, Larisa Gheber

**Affiliations:** 1 Department of Chemistry and Ilse Katz Institute for Nanoscale Science and Technology, Ben Gurion University of the Negev, Beer Sheva 84105, Israel

## Abstract

The *Saccharomyces cerevisiae* kinesin-5 Cin8 performs essential mitotic functions in spindle assembly and anaphase B spindle elongation. Recent work has shown that Cin8 is a bi-directional motor which moves towards the minus-end of microtubules (MTs) under high ionic strength (IS) conditions and changes directionality in low IS conditions and when bound between anti-parallel microtubules. Previous work from our laboratory has also indicated that Cin8 is differentially phosphorylated during late anaphase at cyclin-dependent kinase 1 (Cdk1)-specific sites located in its motor domain. *In vivo*, such phosphorylation causes Cin8 detachment from spindles and reduces the spindle elongation rate, while maintaining proper spindle morphology. To study the effect of phosphorylation on Cin8 motor function, we examined *in vitro* motile properties of wild type Cin8, as well as its phosphorylation using phospho-deficient and phospho-mimic variants, in a single molecule fluorescence motility assay. Analysis was performed on whole cell extracts and on purified Cin8 samples. We found that addition of negative charges in the phospho-mimic mutant weakened the MT-motor interaction, increased motor velocity and promoted minus-end-directed motility. These results indicate that phosphorylation in the catalytic domain of Cin8 regulates its motor function.

Mitotic cell division is an essential process by which duplicate chromosomes are faithfully segregated from mother to two daughter cells. Chromosome segregation during mitosis is mediated by the mitotic spindle, a dynamic bipolar structure composed of microtubules (MTs). MTs filaments are built of αβ tubulin heterodimers and exhibit dynamic instability, manifested by switches between phases of slow growth and fast shrinkage, primarily at their plus end, terminated by the β-subunit.

Several types of MTs are found in the spindle apparatus, such as those MTs that bind chromosomes via a protein complex termed the kinetochore^1^. In addition, two other MT populations exist, namely MTs that point towards the cell cortex and facilitate positioning of the spindle^2^,^3^,^4^ and MTs that overlap in the middle region of the spindle to create an anti-parallel MT array, termed the midzone. Spindle assembly, maintenance of the spindle structure and anaphase spindle elongation are largely achieved by forces applied on the anti-parallel MT array of the midzone^5^.

To properly segregate chromosomes, the mitotic spindle undergoes dynamic morphological changes in each mitotic cycle^6^,^7^. The major molecular factors that mediate these dynamics include kinesin-related motor proteins that utilize energy from ATP hydrolysis to move along MTs (reviewed in^8^). In particular, conserved mitotic kinesin-5 family members have been shown to perform essential functions in mitotic spindle dynamics. Kinesin-5 proteins are unique motors in that they function as homo-tetrameric complexes presenting two pairs of catalytic motor domains located at opposite ends of the active complex^9^,^10^,^11^. This structure allows kinesin-5 motors to crosslink and spread anti-parallel MTs^12^,^13^,^14^. At the spindle, kinesin-5 motors have been shown to localize to the midzone, where, upon sliding anti-parallel MTs apart, they provide the outwardly-directed force to separate the spindle poles (SPBs) and perform essential functions in spindle assembly and maintenance of the bipolar spindle structure^15^,^16^,^17^,^18^,^19^, and in anaphase B spindle elongation^20^,^21^.

The anti-parallel MT-sliding activity at the midzone can only be achieved by the plus-end-directed motility of kinesin-5 motors bound between the anti-parallel MTs^13^,^22^. Indeed, several kinesin-5 motors have been shown to be plus-end-directed *in vitro*^12^,^13^. These finding were in accordance with the accepted dogma holding that the majority of kinesin-related motor proteins with N-terminal motor domains undergoes unidirectional motility towards MT plus-ends. The only kinesin motors that generate minus-end motility belong to the kinesin-14 class, which carry the motor domains in the C-terminus^23^,^24^,^25^. However, it has been recently reported that serval kinesin-5 motors, such as *Saccharomyces cerevisiae* Cin8^26^,^27^ and Kip1^28^ and *Schizosaccharomyces pombe* Cut7^29^, are bi-directional and can move processively towards the MT minus-end. The mechanism and regulation of minus-end-directed and the bi-directional motility of these motors remain unclear.

Cin8, the mitotic *S. cerevisiae* kinesin-5 motor protein homolog, was shown to play important roles in mitosis, in particular in spindle assembly^17^,^22^,^30^,^31^, anaphase B spindle elongation^32^,^33^,^34^,^35^, and kinetochore clustering^36^,^37^,^38^. Previous work had demonstrated that Cin8 exhibits bi-directional motility both in cells and *in vitro*^26^,^27^. On spindles, Cin8 exhibits slow (~26 nm/s) plus-end-directed motility towards the midzone and faster motility (~55 nm/s) in the minus-end direction, towards the SPBs^26^. *In vitro*, under high IS conditions, Cin8 moves fast (102–380 nm/s) and processively towards the minus end of MTs^26^,^27^, yet switches directionality under low IS conditions^26^ and between anti-parallel MTs^26^,^27^. The regulation of the directionality switch of Cin8 remains unclear.

One of the major coordinators of mitotic events is the conserved cyclin-dependent protein kinase 1 (Cdk1, Cdc28 in *S. cerevisiae*). The mitotic functions of Cdk1 are activated before S-phase and persist throughout mitosis by association with the accessory mitotic B-type cyclins (Clb1-6 in yeast). The levels of the six Clb cyclins oscillate^39^, thus providing stage-specific functions for Cdk1^40^. It has been previously shown that Cin8 is regulated by phosphorylation of at least one of the Cdk1-specific sites located in its catalytic domain, i.e. S277, T285 and S493^30^,^41^ (Fig. 1a). S493 is located between β-sheet 8 and α-helix 6 of the kinesin motor domain and is highly conserved among kinesin family members (Fig. 1a)^42^. Based on the structure of HsEg5^43^,^44^, S493 is located in proximity to the kinesin-motor ATP-binding loop (P-loop). Residues S277 and T285 are located in the loop 8 of Cin8, which is the longest loop 8 among kinesin family members and is not conserved among kinesin-5 motors of higher eukaryotes. Interestingly, S277 is conserved among fungal kinesin-5 homologs which contain inserts in loop 8 that are longer than those found in kinesin-5 motors from higher eukaryotes (Fig. 1a). On the other hand, T285 is not conserved and, instead, is unique to Cin8. *In vivo*, these three sites are differentially phosphorylated in late anaphase^41^. Using phospho-deficient and phospho-mimic mutants, it has been demonstrated that detachment of Cin8 from the spindle in anaphase is dependent on Cin8 phosphorylation since in contrast to wild type (wt) and phospho-deficient Cin8, the phospho-deficient Cin8 mutant failed to detach from the spindle in mid-late anaphase^41^. It had also been demonstrated that phosphorylation-dependent detachment of Cin8 from the spindle is required to slow down the spindle elongation rate and to maintain correct spindle morphology^41^.

To examine whether Cin8 phosphorylation regulates its motile properties and directionality, in this study we examined the motility of wt Cin8 and its phospho-mutants using a single molecule fluorescence motility assay. Measurements were performed on whole cell extracts, as well as on purified samples, in several IS conditions. Our results indicate that phosphorylation of Cdk1-specific sites within the Cin8 catalytic domain affects motor-MT interactions and alters Cin8 velocity and directionality. These results suggest that phospho-regulation of the intracellular functions of mitotic kinesins involves regulation of their motile properties.

## Results

### Bi-directional movement of Cin8 variants is ATP-dependent

**Figure 1:**
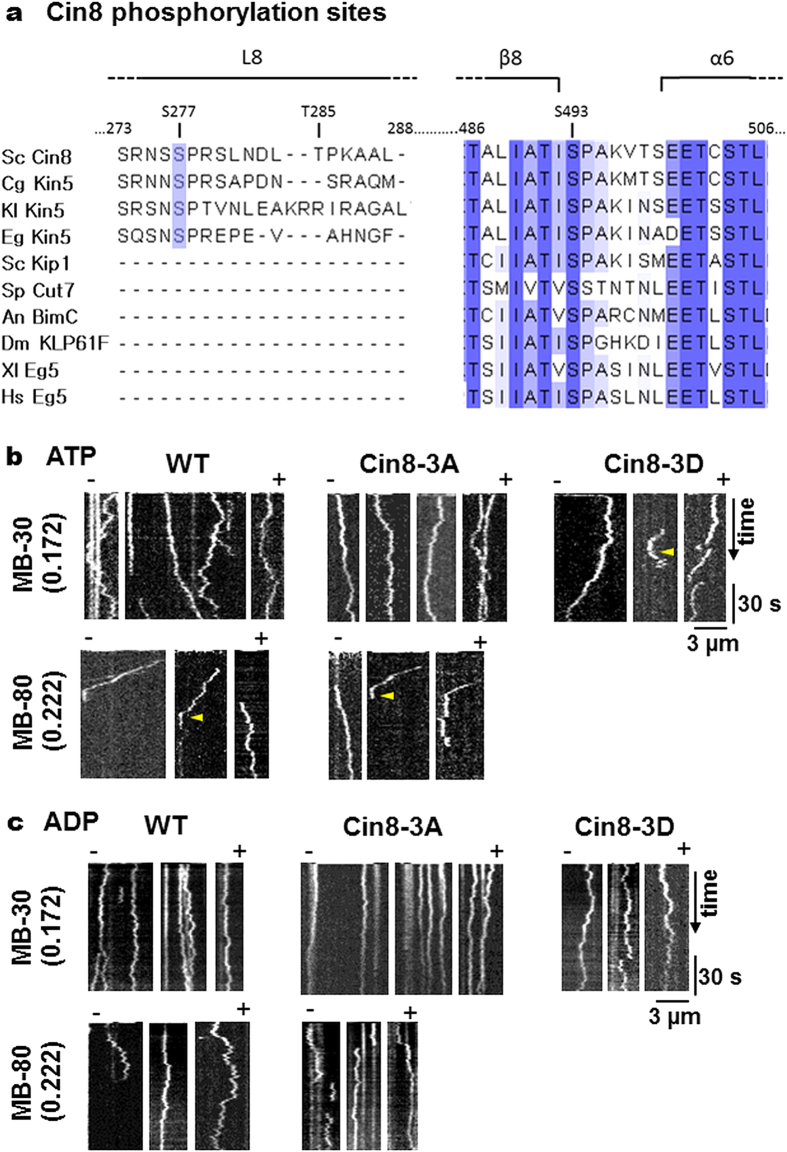
Cin8 phosphorylation sites and representative kymographs of the movements of Cin8 phospho-variants in whole cell extracts. (**a**) Multiple sequence alignment of Cdk1-specific sites in the catalytic domain of Cin8. Secondary structure elements are marked on the top, based on the kinesin super-family motor domain amino-acid alignment, and compared to the alignments derived from the crystal structures of HsKHC and DmNcd^42^. Kin5: kinesin-5; Sc: *Saccharomyces cerevisiae*; Cg: *Candida glabrata*; Kl: *Kluyveromyces lactis*; Eg: *Eremothecium gossypii*; Sp: *Schizosaccharomyces pombe*; An: *Aspergillus nidulans*; Dm: *Drosophila melanogaster*; Xl: *Xenopus laevis*; Hs: *Homo sapiens*. (**b**,**c**) Experiments were performed using extracts of cells expressing the three variants (indicated on top) in the presence of 4 mM ATP (**b**) or 4 mM ADP (**c**). Buffer and IS conditions are indicated on the left (MB-30: MB with 30 mM NaCl added; MB-80: MB with 80 mM NaCl added). Measurements were performed on polarity-marked MTs, with the minus-ends being on the left of each kymograph, as indicated on the top. Yellow arrowheads indicate detachment of Cin8 from the MTs.

To examine the effect of Cdk1-mediated phosphorylation on Cin8 motility, we analyzed the motile properties of three Cin8 variants, namely wt Cin8, phospho-deficient Cin8-3A, in which the serines and threonine in Cdk1-specific sites within the Cin8 catalytic domain were mutated to alanine (S277A, T285A, S493A), and the phospho-mimic, Cin8-3D mutant, in which the same amino acids were mutated to negatively-charged aspartic acids (S277D, T285D, S493D). These variants were examined in crude extracts of cells expressing Cin8 under its own promoter, integrated into the genome. *In vitro* single molecule motility measurements were performed under two IS conditions, i.e. low IS (motility buffer (MB) supplemented with 30 mM NaCl, IS = 0.172) and intermediate IS (MB supplemented with 80 mM NaCl, IS = 0.222) on polarity-marked MTs. Representative kymographs obtained from time-lapse recordings of single molecule motility events of Cin8-3GFP variants are shown in Fig. 1b (supplementary movies S1–S3). Only two very short movements where observed for Cin8-3D in the intermediate IS (not shown).

**Figure 2:**
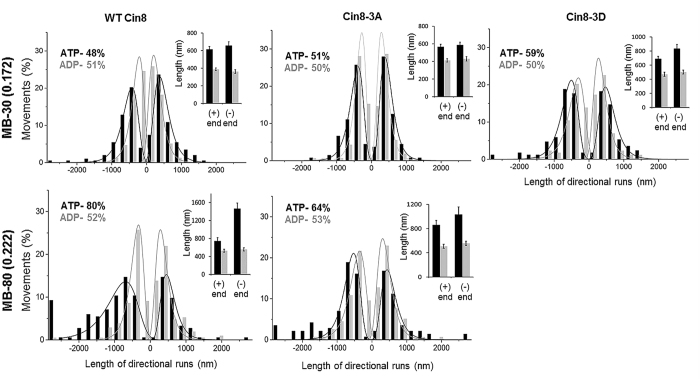
Length of directional runs of Cin8 phospho-variants in whole cell extracts in the presence ATP or ADP. Histograms of the lengths of directional runs obtained from kymographs (Fig. 1b,c) of directional runs of Cin8 variants. The included runs were at least 2 pixels (252 nm)-long, with stall periods of less than 15 s and for which the beginning and end of the run were observed in the kymograph. IS conditions are indicated on the left. Black: ATP; gray: ADP. In the upper right corner of each panel, the bar graph presents the average ± SEM of the lengths of directional runs in the plus- and minus-end directions. In all cases, there is a significant difference (P < 0.001) between the average length of directional runs in the presence of ATP or ADP. The percentage of directional runs in the minus-end direction is indicated on the left of each panel. The number of runs in each histogram is between 136 and 203.

To examine whether the bi-directional motility of Cin8 variants is of a diffusive nature^45^, we compared the motility of Cin8 variants in whole cell extracts, in the presence of ATP (Fig. 1a, supplementary movies S1–S3) or ADP (Fig. 1c, supplementary movies S4–S6). Histograms describing the lengths of directional runs (see Materials and Methods) that occurred in the presence of both nucleotides are presented in Fig. 2. In all cases, we observed a significant increase in the length of directional runs in the presence of ATP, as compared to ADP (Fig. 2). Furthermore, In agreement with previous reports for Cin8 and Kip1^26^,^28^, in the presence of ATP, an increase in IS caused a shift of motility direction toward the minus-end of the MTs in the cases of the wt and Cin8-3A variants, a similar bias towards the minus-end can also be seen for cin8-3D in low IS conditions (Fig. 2). On the other hand, no directionality bias in the presence of ADP was observed in any case, with ~50% of movements being in both directions, indicating diffusive motility (Fig. 2). Therefore, bias towards minus-end directionality and the longer average lengths of directional runs that took place in the presence of ATP indicate that these movements have a directional component and are not merely diffusive.

### Velocity analysis of bi-directional Cin8 movement

To analyze the velocities of Cin8 phospho-variants, we first examined the net velocity of motility episodes throughout the total interaction time of Cin8 with the MTs, termed here as “full runs” (Table 1). These measurements were performed based on kymographic representation of Cin8 movements (see Materials and Methods). The average velocity values of full runs of Cin8 were calculated by assigning a “weight” to each run equal to the duration of the run. The average velocity and the standard error of the mean (SEM) were calculated using equations 1 and 2 (see Materials and Methods). The results indicated that in low IS buffer in the presence of ATP, the Cin8-3D variant exhibited a higher percentage of movements in the minus-end direction, as compared to Cin8 and the Cin8-3A variants, which resulted in a net negative velocity of Cin8-3D (Table 1). The increase in percentage of the minus-end directed motility could also be observed in high IS buffer for Cin8 and the Cin8-3A variant. However, the increase in minus-end motility events could not be seen in the presence of ADP, indicating that the minus-end directed motility bias in higher IS buffer and of the Cin8-3D variant is ATP-dependent.

**Figure 3:**
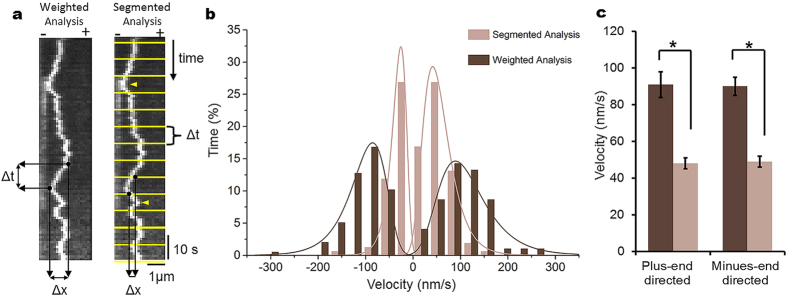
Comparison of velocity analysis methods. (**a**) Left: Weighted analysis of directional runs. Measurement is from start to end of a unidirectional movement (run). Right: Segmented analysis. The kymograph is divided into pre-determined time intervals (yellow lines) and measurements are performed between the two yellow lines. Yellow arrowheads point to movement directional changes within a segment. (**b**) Velocity distribution of the same data set obtained by the two methods. Measurements of wt Cin8 in a whole cell extract in MB-30 buffer and 4 mM ATP are shown (see Fig. 1b, top left). Segmented analysis was performed on 6 s segments. The weighted analysis histogram was constructed according to equation 3 (see Materials and Methods). The histograms reflect 196 movements and 160 segments. Percentages of total movement time in the minus-end direction are 48% for the weighted analysis and 49% for the segmented analysis. (**c**) Columns and bars represent averages ± SEM of velocity distributions shown in panel B. Weighted averages ± SEM were calculated using equations 1 and 2, respectively (see Materials and Methods), while for segmented analysis, such values correspond to a simple average ± SEM. *P < 0.005.

The values of the net velocity obtained from analysis of the full runs of Cin8 (Table 1) were considerably lower than values reported previously by us for wt Cin8^26^. This is likely due to the averaging of stall periods and movements in the opposite directions that occurred within a single full run (Fig. 1b). Therefore, to determine velocity values that reliably represented the motility of Cin8, we analyzed the velocity of Cin8 via two alternative methods (Fig. 3). We previously used the first method, referred to as “segmented” analysis, for analysis of Cin8 and Kip1 motility^26^,^28^. According to this method, the kymograph of a continuous movement is divided into equal time segments and the velocity of each segment is determined by assessing the coordinates of the start and end points of the movement in each segment (Fig. 3a, right panel). We initially used this method since it assigns higher weight for velocities of longer directional runs^28^,^41^. However, this method introduces bias towards slow velocity values since it includes movements with changes in motility direction within a particular time segment (Fig. 3a, right panel, yellow arrows). In addition, there is a degree of freedom in determining the time interval of the segments, which can also affect the results. To address these problems, we instead considered a method referred to as “weighted” analysis of directional runs/movements of Cin8. According to this method, displacement of two pixels (252 nm) or more in the same direction on a kymograph was assigned as a new directional run (see Materials and Methods). The velocity of the directional run was determined by assessing the coordinates of its start and end points (Fig. 3a, left panel). If a stall period of less than 15 s observed between two run in the same direction, it was still considered as one directional run. Next, a “weight” of each directional run was allocated, equal to the duration of the run, which provided a time-weight value of each directional run. This procedure is similar to “weight” allocation for the full runs of Cin8. The average velocity, the standard error of the mean (SEM) and the velocity distribution were calculated through the use of equations 1, 2 and 3, equations 2 and 3 were developed for this study (see Materials and Methods).

To compare the two methods, we determined the velocity distributions and averages ± SEM were obtained by both using the same data set of time-lapse recordings of wt Cin8 motility on polarity-marked MTs in MB-30 buffer containing 4 mM ATP (Fig. 1b, top left). Under these conditions, the motility of Cin8 was previously shown to be bi-directional^26^. Our results show that the percentages of plus- and minus-end-directed runs provided by both analyses were similar (Fig. 3b). However, the velocity distribution obtained by segmented analysis was considerably narrower, as compared to the values obtained by weighed analysis (Fig. 3b). As a result, average velocities in the plus- and minus-end directions were ~45% lower when obtained using segmented analysis, as opposed to weighed analysis (Fig. 3c). This discrepancy is likely due to the fact that weighted analysis eliminates the low-velocity bias introduced by segmented analysis. Since weighted analysis also takes the duration of directional runs into account and assigns higher weights to velocities of longer runs, we believe that weighted analysis represents better the bi-directional motility of Cin8 and thus, was used in this study.

### Phosphorylation of the Cin8 catalytic domain regulates its attachment to MTs

The three sites of Cdk1-mediated phosphorylation present in the catalytic domain of Cin8 (S277, T285 and S493) were previously shown to affect Cin8 localization and attachment to the mitotic spindle^41^. To examine the effect of Cdk1-mediated phosphorylation on the interaction of Cin8 with MTs, we analyzed motility of three Cin8 variants collected in two IS conditions (Fig. 1b, supplementary movies S1–S3). Based on the slopes of the movements recorded in the kymographs, we found that in higher IS conditions, Cin8 motility was faster and more minus-end-directed, as compared to motility in low IS conditions, consistent with our previous findings^26^. We also found that increasing IS caused a decrease in the percentage of moving Cin8 molecules attached to MTs throughout the 90 s duration of our measurement. For example, at IS = 0.172 M, 75% of wt Cin8 molecules attached to the MTs for longer than 90 s, while only 21% remained attached at IS = 0.222 M. A similar trend was observed for the other Cin8 variants (Fig. 1b, yellow arrowheads; Table 2, extract). These results indicate that increasing IS weakened interactions between Cin8 and MTs, thus increasing detachment of single Cin8 molecules and shortening their interaction time with MTs. These effects were likely to have been caused by a masking of the electrostatic interactions between Cin8 and the MTs in higher IS conditions. Interestingly, the same effects were also observed when comparing the behavior of phospho-mimic Cin8-3D mutant with that of the wt and phospho-deficient Cin8-3A variants. Reminiscent to what was observed upon increasing IS, the Cin8-3D variant exhibited higher velocity and increased minus-end directionality, as compared to Cin8 and Cin8-3A (Fig. 2a, upper panels, supplementary movie S3). In addition, the percentage of molecules moving longer than 90 s was significantly lower under all conditions for the Cin8-3D variant, as compared to Cin8 and Cin8-3A (Fig. 1b, yellow arrowheads; Table 2, extract). These results indicate that the affinity of the phospho-mimic mutant Cin8-3D to MTs was significantly lower, as compared to that of the wt and Cin8-3A variants.

**Figure 4:**
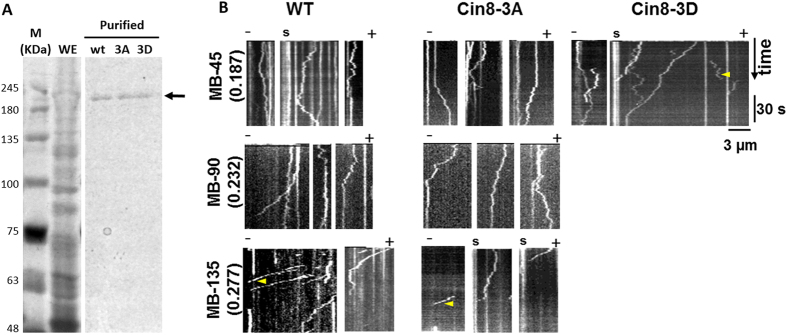
Representative kymographs of movements of purified Cin8 variants. (**A**) Purification of Cin8 phospho-variants. Cin8 samples were fractionated on 7.5% SDS acrylamide gels and stained using Coomassie brilliant blue. Cin8 variants were purified on a nickel affinity column followed by a size-exclusion chromatography. Arrows indicate the expected molecular weight of Cin8-3GFP-6His; WE: whole cell extract; M (kDa): molecular weight marker; wt: wild type Cin8; 3A: Cin8-3A; and 3D: Cin8-3D. (**B**) Representative kymographs of movements of purified Cin8 variants (indicated on top) in the presence of 4 mM ATP. Buffer and IS conditions are indicated on the left. Measurements were performed on polarity-marked MTs, with the minus-ends being on the left of each kymograph, as indicated on top. In some cases, the minus-end seeds of the MTs were marked with Atto-488-labeled tubulin and can be seen in the GFP channel. This is indicated with the letter **s**. Yellow arrowheads highlight detachment of Cin8 from the MTs. We could not detect movement of Cin-3D in intermediate (MB-90) or high (MB-135) IS conditions.

To examine if these phenomena are intrinsic to Cin8 or dependent on other proteins in the whole cell extracts, we examined the motile properties of purified wt Cin8 and its two phospho-mutants. Accordingly, Cin8 variants were over-expressed in *S. cerevisiae* using the *pGAL* promoter on a 2-micron plasmid, and purified using nickel affinity followed by a size-exultation chromatography (see Materials and Methods). The procedure yielded pure proteins (Fig. 4A) that were used in *in vitro* motility assays under three IS conditions, low IS (MB supplemented with 45 mM NaCl, IS = 0.187), intermediate IS (MB supplemented with 90 mM NaCl, IS = 0.232); and high IS (MB supplemented with 135 mM NaCl, IS = 0.277). Representative kymographs obtained from time-lapse recordings of single molecule motility events of the purified Cin8-3GFP variants are shown in Fig. 4B (supplementary movies S7–S9).

Similar to the whole cell extract, increased IS caused an increase in the velocity of minus-end-directed motility of purified wt Cin8 and Cin8 3A, as indicated by steeper slopes in the kymographs (Fig. 4b). We also observed a decrease in number of purified Cin8 molecules that remained attached to MTs longer than 90 s with increasing IS (Fig. 4B, yellow arrowheads; Table 2, pure). Finally, similar to whole extracts samples, the purified Cin8-3D variant exhibited faster minus-end directed motility and more frequent detachments from the MTs compared to wt and Cin8-3A (Fig. 4b, yellow arrowheads; Table 2, pure). We could not detect any movement of Cin8-3D in intermediate (MB-90) of high (MB-135) IS conditions, an observation that is consistent with the notion that the affinity of the phospho-mimic Cin8-3D variant to MTs is significantly reduced under these conditions.

### Quantitative assessment of the velocity and directionality of Cin8 phospho-variants

**Figure 5:**
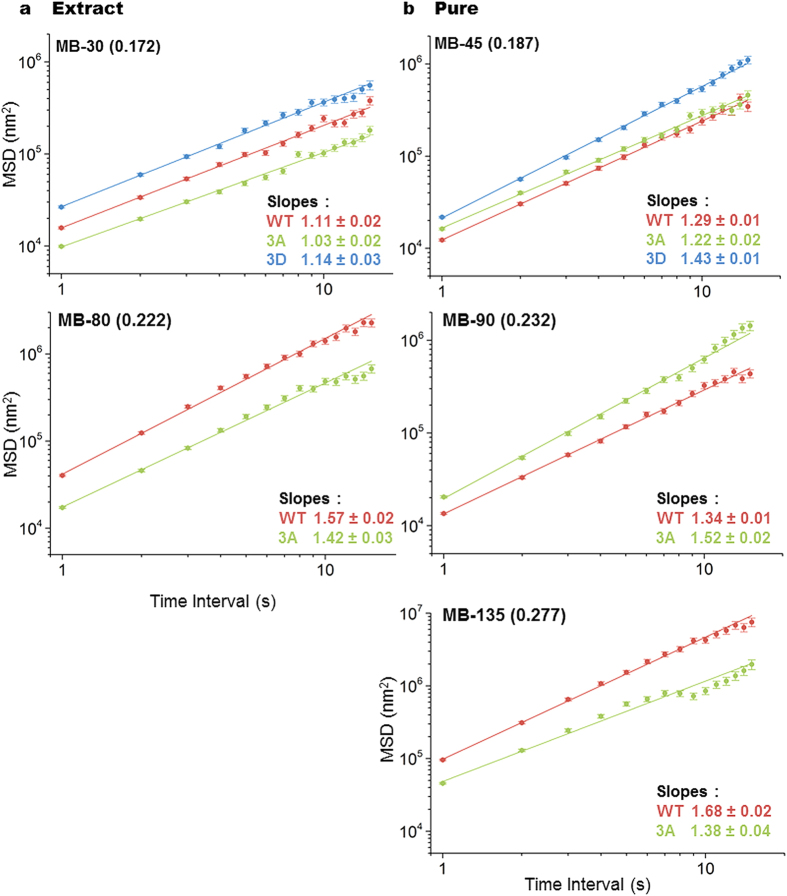
MSD analysis. MSD analysis of Cin8 movements was performed by tracking the location of single Cin8 molecules, using the ImageJ Spot Tracker plug-in. MSD values were only taken for movements longer than 630 nm, and with a stall time of less than 15 s. The results of MSD analysis versus time intervals were plotted on a log-log scale and fitted to a liner equation. In all panels, wt Cin8 – red, Cin8-3A – green, Cin8-3D –blue. Buffer and IS conditions are indicated at the top left and the slopes ± SEM are indicated at the bottom right. (**a**) Crude extracts. (**b**) Pure samples.

We next quantitatively examined the effect of mutations in the sites of Cdk1-mediated phosphorylation in the Cin8 catalytic domain on the velocity and directionality of the protein. To omit movements that might result from pure diffusion in such analysis, we considered only those movements that were 630 nm (5 pixels) in length or longer. This limit was obtained by assessing the motility of Cin8 variants in the presence of ADP (Figs 1c and 2). The longest average directional run in the presence of ADP was 556 ± 35 nm (n = 110) for wt Cin8 at MB-80 in the minus-end direction. Therefore, setting a limit of 630 nm eliminated the majority of purely diffusive motility events. To determine whether the movements analyzed following the elimination of short movements were not diffusive, we performed mean square displacement (MSD) analysis on data obtained from all movement trajectories with directional runs longer than 630 nm and presenting stall times of less than 15 s (see Materials and Methods). The results MSD analyses were plotted versus the time interval in log-log scale and fitted linearly (Fig. 5). In all cases but one (i.e. the whole cell extract containing Cin8-3A measured in low IS buffer), we obtained a slope that was significantly higher than 1 (in most cases, considerably higher). This result indicates that Cin8 motility has a directed motility component and is not merely diffusive^46^,^47^.

**Figure 6:**
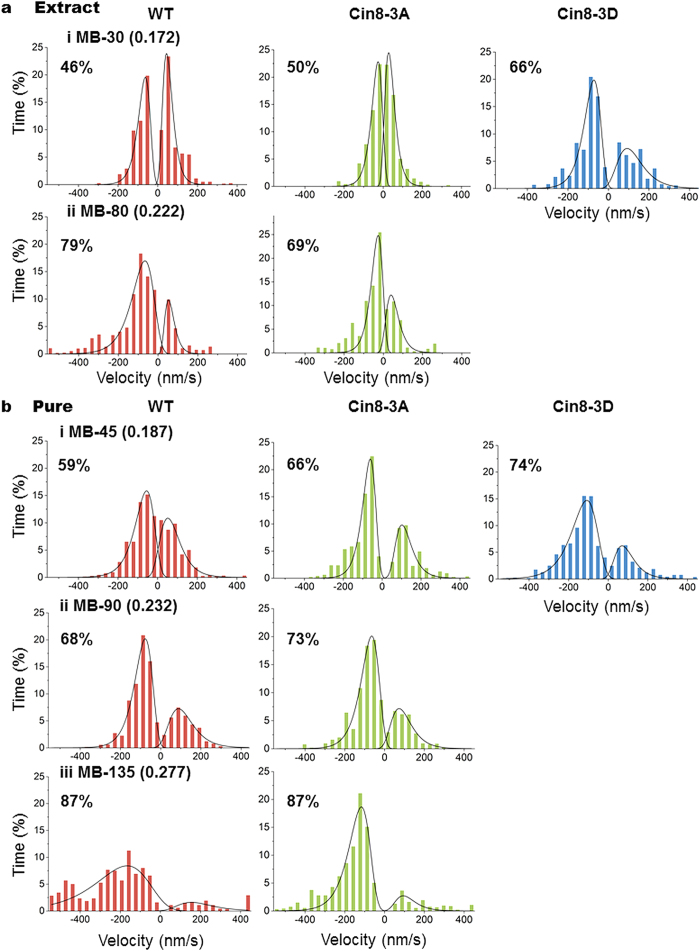
Velocity distribution of directional runs of Cin8 phospho-mutants in different IS conditions. Distributions were constructed after weighted analysis of movements of the motors in kymographs, using equation 3 (see Materials and Methods). Each histogram was built from results obtained from 674-2163 s of movement (after discarding movements shorter than 630 nm) in crude extracts (**a**) and pure (**b**) samples. Cin8 variants are indicated on the top. Buffer and IS conditions are indicated at the left of each row. The percentage of total movement times in the minus-end direction of the MTs is indicated in the upper left corner of each panel.

**Figure 7:**
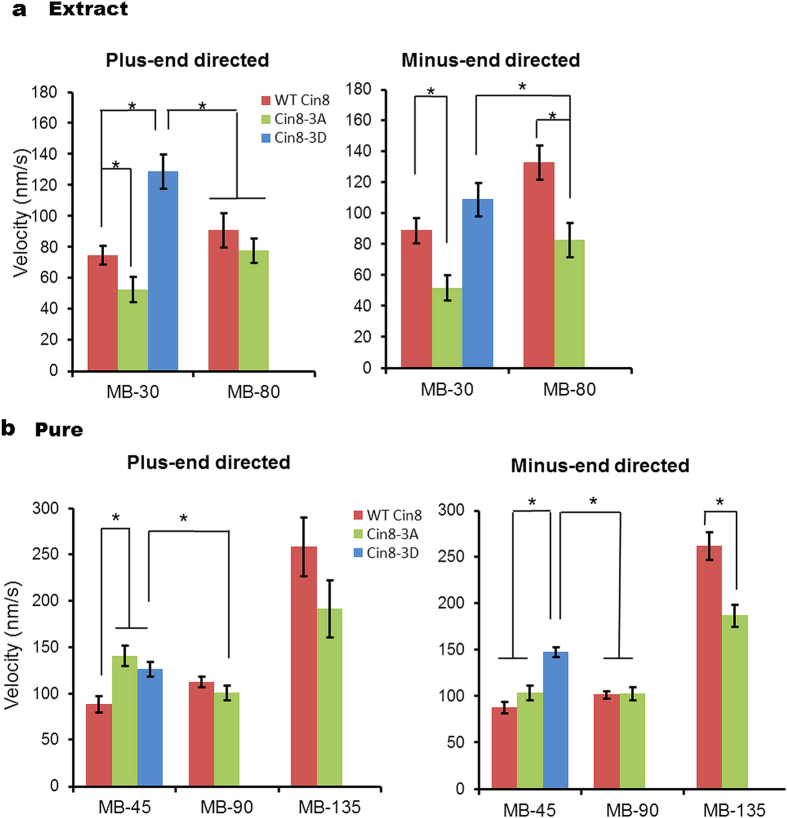
Average velocities of directional runs of Cin8 phospho-variants in different IS conditions. Columns and bars represent weighted averages ± SEM of the velocity distributions shown in Fig. 6. The weighted average ± SEM were calculated using equations 1 and 2, respectively (see Materials and Methods). The examined variants are: wt Cin8 – red; Cin8-3A – green and Cin8-3D –blue, examined in crude extracts (**a**) or as pure samples (**b**). *P < 0.005.

We next determined velocity distribution in the kymographs using the weighted analysis method, including only those directional runs longer than 630 nm (Fig. 6). We observed that increasing IS broadened the velocity distribution for all variants, and increased the velocities in both the minus- and plus-end directions of the MTs (Fig. 6). A comparison of velocity distribution of the Cin8 variants at the same IS indicated that in most cases, the velocity destitution of Cin8-3A was the most narrow and with the slowest velocities, while the velocity distribution of Cin8-3D was the widest and with the highest velocities. A comparison of average velocities for all conditions (Fig. 7) revealed that in most cases, when there was a significant difference between the average velocities of the variants, the motility of Cin8-3D was fastest, as compared to the wt Cin8 and Cin8-3A variants. Moreover, in most cases Cin8-3D in low IS was even faster than wt Cin8 and Cin8-3A in intermediate IS (Fig. 7) indicating a substantial effect of phosphorylation on Cin8 velocity. In addition, the velocity of wt Cin8 was higher than that of Cin8-3A in most cases. The only exception noted was obtained upon assessment of plus-end-directed velocity using pure samples at low IS (Fig. 7b, left), although this may have resulted from the relatively small number of measurements taken under such conditions (n = 42). Thus, we determined the average velocity ranking of Cin8 variants to be Cin8-3D > Cin8 > Cin8-3A.

Consistent with a previous report^26^, the percent of minus-end-directed runs was influenced by the IS of the buffer, with higher IS inducing augmented minus-end-directed motility. This trend was apparent for all Cin8 variants examined, be they purified or crude extract samples (Fig. 6). In the same IS conditions, differences in the percentage of runs in the minus-end direction were observed among the Cin8 variants. For purified samples and in whole cell extracts under low IS conditions, Cin8-3D was more minus-end-directed, as compared to wt Cin8 and Cin8-3A (Fig. 6a,b, upper panels). These results indicate that similarly to the effect of increasing IS, the addition of negative charges upon phosphorylation at Cdk1-specific sites within the Cin8 catalytic domain led to increased minus-end-directed motility.

## Discussion

The results presented here indicate that mutations in Cdk1 phosphorylation sites within the motor domain of Cin8 affect its motile properties. First, the phospho-mimic mutant, Cin8-3D, had lower affinity to MTs, as compared to wt Cin8 and Cin8-3A, indicated by its increased detachment from MTs (Figs 1b and 4b, Table 2). Second, phosphorylation affected the Cin8 velocity, causing it to exhibit a wider velocity distribution (Fig. 6) and significantly higher average velocity (Fig. 7). Most importantly, phosphorylation promoted minus-end-directed motility, as manifested by the longer time spent by Cin8 molecules moving in the minus-end direction (Fig. 6.). Since all these effects are comparable to the effect of increasing the IS of the buffer (Figs 6 and 7, Table 2), we concluded that the effect of phosphorylation on Cin8 motility is electrostatic in nature. The calculated net charge at pH 7 of the catalytic domain of Cin8 and β-tubulin are +13 and −31, respectively. Therefore, the effects we report here can be explained, in part, by differences in charge of Cin8 and MTs at physiological pH. Phosphorylation within the Cin8 catalytic domain, i.e. addition of negative charges to this positively charged Cin8 domain, weakens interactions between Cin8 and negatively-charged MTs. This is likely to increase the rate of detachment of the motor from the MTs. Similarly, increased IS masked the charge attraction between Cin8 and MTs, likely weakening MT-motor interactions. This increased the detachment of wt Cin8 and Cin8-3A from MTs and abolished the already weakened interaction of Cin8-3D with these cytoskeletal components (Figs 1b and 4b, Table 2). These results agree with the effect of charge addition on MT-motor interactions for kinesin motor family proteins, as previously reported for Kinesin-1^48^, where decreasing the levels of positive charge at the MT-motor interaction site weakened the interaction between the two. Consistently, it was also demonstrated that decreasing the degree of negative charges in MTs significantly reduced the affinity of the MTs to kinesin^49^. The atomic structure of Cin8 has not yet been determined. However, two of the three Cdk1-modified sites that we have examined, S277 and T285, are located within the large Cin8 loop 8 insert (Fig. 1a), which was shown for other kinesin motors to face the MT lattice in the MT-motor complex 50,51. Thus, it is likely that changing the charge of these residues would affect the interaction of Cin8 with MTs.

In addition to affecting the interaction between Cin8 and MTs, phosphorylation can influence Cin8 motor function by an alternative mechanism(s). For example, phosphorylation can induce conformational changes, such as in the case of kinesin-13 MCAK, where phosphorylation induces a conformation change in the entire protein, such that interactions with MTs are inhibited^52^.

We observed that in most cases, the ranking of velocity for the Cin8 variants in the same conditions was Cin8-3D > Cin8 > Cin8-3A (Figs 6 and 7). This rank is consistent with degree of the negative charge/dipole of S/T residues within the Cdk1-modified sites of the three variants; the more negatively charged are the residues, the higher is the velocity. Thus, a possible explanation for the differences in the average velocity of the variants considered is that the reduced electrostatic interaction between the motor and MTs is caused by negative charges added upon phosphorylation. Consistent with this notion is the fact that a masking of electrostatic interactions by increasing the IS also increased Cin8 velocity, (Figs 6 and 7 and ref. 26). The weakening of the Cin8-MT interaction which resulted in faster movement due to the faster release of one motor domain from the MTs is also likely to increase the probability of release of both motor heads from the MTs, resulting in Cin8 detachment. Therefore, the correlation between velocity and probability of detachment from MTs reported here is likely to represent a general property of kinesin motors. Although the rate-limiting step of the kinesin-5 catalytic cycle is still under debate, it has been reported that the dissociation of the motor head from MTs following ATP hydrolysis^53^,^54^ is the slowest step. Hence, reducing the motor-MT interaction by phosphorylation might accelerate this step, thereby accelerating the overall catalytic cycle and resulting in faster motility. In other cases, it has been reported that ATP hydrolysis is the rate-limiting step^55^. Thus, an alternative possibility is that the phosphorylation of Cin8 Cdk1 site(s) also influence the ATP hydrolysis. Based on multiple sequence alignment and the known structure of the human kinesin-5 homolog HsEg5^43^,^44^ (Fig. 1a), the conserved phosphorylation site, S493, is localized close to the motor P-loop. It is possible that phosphorylation of S493 by Cdk1 changes the micro-environment in the ATP-binding pocket, affecting the rate of the ATP hydrolysis, resulting in faster motility. Further studies are needed to examine the effect of phosphorylation on the Cin8 ATPase cycle and to distinguish between the possibilities listed above.

We have previously reported that localization of Cin8 to anaphase spindles is regulated by phosphorylation in the catalytic domain of this motor^41^. Using the same phospho-variants as employed here, we demonstrated that the Cin8-3D mutant detached from the mitotic spindle, whereas the Cin8-3A mutant remained attached throughout the duration of anaphase. Thus, our *in vitro* findings are in agreement with our previous *in vivo* results, indicating that phosphorylation in the Cin8 motor domain causes its detachment from spindle MTs during anaphase^41^ by directly regulating its motile properties. There are limited reports of phosphorylation in the catalytic domain of kinesin-related motor proteins directly affecting their motility. A similar finding was reported for the *Caenorhabditis elegans* kinesin-6 homolog ZEN-4, in which phosphorylation of the motor domain by Cdk1 reduced the affinity of ZEN-4 for MTs^56^. It was also demonstrated that phosphorylation of the conserved serine residue in the motor domain of the *Drosophila melanogaster* kinesin-13 KLP10A altered its interaction with the MTs and diminished its MT-depolymerizing activity^57^. Thus, results obtained in the *in vitro* experiments described here are directly relevant to the *in vivo* functions of the motor and are likely to reflect a general mechanism for regulating motor function. Furthermore, the similar results we obtained with purified proteins and whole cell extracts (Figs 1b and 4b, Table 2) suggest that this regulatory mechanism is an intrinsic property of Cin8 and is largely independent of cellular factors.

Thus far, three kinesin-5 motors have been reported to be bi-directional^26^,^27^,^28^,^29^, suggesting that the minus-end-directed and bidirectional motility of kinesin-5 motors is of physiological importance. Our finding, reported here for the first time, strongly suggests that the intracellular cell-cycle regulatory machinery governs directionality of kinesin-5 motors. Although the exact role of the minus-end-directed motility still remains unclear, we can hypothesize that since phosphorylation of Cin8 occurs at mid-late anaphase^41^, increasing minus-end-directed motility at this stage would serve to clear Cin8 from the midzone region, directed the motor towards the spindle poles, thereby slowing the spindle elongation rate and facilitating spindle breakdown at the end of anaphase.

To summarize, by using phospho-deficient and phospho-mimic Cin8 mutants, we found that phosphorylation significantly affects the motile properties of this motor protein. Phosphorylation in the Cin8 motor domain reduced motor-MT interactions, inducing detachment from MTs, increasing motility velocity and promoting minus-end-directed motility. We propose that these effects represent a general mechanism for regulating mitotic kinesin-related motor proteins.

## Materials and Methods

### Yeast strains and sample preparation

The *S. cerevisiae* strains and plasmids used in this study are described in Table 3. For whole cell extracts, 0.5–2 L of cell culture were grown in minimal medium to mid-log phase, pelleted and washed once with purified water and twice with MB-175 (175 mM NaCl, 2 mM EDTA, 1 mM EGTA, 10% glycerol, 50 mM Tris-HCl, 30 mM PIPES/KOH, pH 7.2)^12^. The washed pellet was ground with a mortar and pestle in liquid nitrogen containing MB-175 buffer supplemented with complete protease inhibitor (Roche), 0.1% Triton X-100, and 0.1 mM ATP. The ground cells were thawed and centrifuged at 13,000 g for 20 min at 4 °C. The clear supernatant was collected, aliquoted and snap-frozen and stored at −80 °C until use. Purification of Cin8 variants: plasmids for Cin8 overexpression were constructed using standard methods and transformed into a protease deficient yeast strain (Table 3). 2.5–4 L of culture was grown in minimal medium supplemented with 2% raffinose to mid-log phase. For induction of Cin8 expression, 2% galactose was added and the culture was grown for an additional 5 h. Cells were pelleted, washed once with purified water and twice with lysis/binding buffer (500 mM NaCl, 10% glycerol, 2 mM β-ME, 1 mM PMSF, 1 mM MgCl_2_, 0.1 mM ATP, 0.2% Triton X-100, complete protease Inhibitor (Roche), 50 mM Tris-HCl, pH 8) and ground with a mortar and pestle in liquid nitrogen. The ground cells were thawed and centrifuged at 13,000 g for 20 min at 4 °C. Ni-NTA beads (Invitrogen) were added to the cell extract, and binding ensued for 1.5 h on a shaker at 4 °C. The mix was loaded onto a column and washed with 3–5 column volumes of wash buffer (500 mM NaCl, 30 mM imidazole, 10% glycerol, 1.5 mM β-ME, 0.1 mM Mg-ATP, 1 mM PMSF, 0.2% Triton X-100, complete protease Inhibitor (Roche), 50 mM KH_2_PO_4_, pH 8). The protein was eluted with 2–5 ml of elution buffer (500 mM NaCl, 350 mM imidazole, 10% glycerol, 1.5 mM β-ME, 0.1 mM Mg-ATP, 1 mM PMSF, 0.2% Triton X-100, complete protease Inhibitor (Roche), 50 mM KH_2_PO_4_, pH 8). The eluent was collected in 200–500 μl fractions and aliquots were taken for SDS-PAGE analysis to determine the fraction of the eluted protein. The selected fractions were combined and concentrated to a volume of 0.5 ml using a centrifugal concentrating unit with a 100 kDa cutoff. The sample was then loaded onto a Superose 6 10/300 GL column (GE Healthcare) at 4 °C that was previously equilibrated with gel filtration buffer (500 mM NaCl, 0.1 mM MgCl_2_, 0.1 mM ATP, 10 mM DTT, 30 mM Pipes, 50 mM Tris-HCl, pH 7.2) for separation by FPLC. Fractions (0.5 ml) were collected, 15% glycerol was immediately added to each, which were then snap-frozen and stored at −80 °C. Tubulin was purified form porcine brain using a high molarity salt buffer method^58^. SDS-PAGE analysis were performed using standard techniques^59^. Cin8 samples were fractionated on 7.5% SDS acrylamide gels.

### *In vitro* motility assays

*In vitro* motility assays were performed following procedures we previously described^26^,^28^,^60^ in MB of the desired salt concentration supplemented with 10 μM Taxol, 0.1 mg/ml glucose oxidase, 0.8 mg/ml catalase, 1 mM DTT, 10 mM glucose, 0.2 mg/ml casein, 4 mM MgCl_2_, and 4 mM ATP or ADP. MTs were polymerized with TMR-labelled tubulin (TL590M Cytoskeleton Inc.), and polarity marked using Atto-488-labelled seeds or seeds with a significantly higher concentration of TMR-labelled tubulin marking the MT minus-end. Single molecule fluorescence data were collected on a Zeiss Axiovert 200 M-based microscope setup equipped with an HBO 100 Mercury Illuminator, and a cooled CCD camera (SensiCam, PCO or Andor Neo sCMOS) with frame time of 1 s. Data was processed using ImageJ and MetaMorph (MDS Analytical Technologies) software.

### Data analysis

Kymographs were created using MetaMorph software. The net velocity of full runs was calculated for the entire observed period of MT-motor interaction for each motility event. If attachment or detachment of the motor was not observed in a given kymograph, the velocity was calculated for the full duration of our measurement, i.e., 90 s. Since every full run was of different duration, we determined average velocity and SEM by the “weighted” method, in which each full run was assigned a time-weight equal to its duration. The velocity average was calculated according to equation (1):


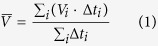


where V_i_ is the velocity of a full run and Δt_i_ is the corresponding duration. The SEM was calculated according to equations (2):


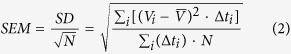


where V_i_ is the velocity of a run, Δt_i_ is the corresponding duration, 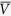
is the average velocity, N is the number of movements, and SD is standard deviation.

For segmented analysis, a grid of horizontal lines 6 pixel apart was overlaid on the kymograph by the grid plug-in tool of ImageJ. The grid served to divide the kymograph into equal time-segments, from which the velocity of each segment of continuous movement was determined^26^,^28^ (Fig. 3a, right). Since all segments were of equal time, each velocity had the same weight. Velocity averages ± SEM and velocity distribution were calculated using standard methods.

For weighed analysis of directional runs, the coordinates of the starting and ending points of a directional run were assigned. A directional run was defined as any unidirectional movement of more than 2 pixels (252 nm). Directional changes of one pixel (126 nm) were not taken into account. A directional change of two pixels or more was defined as a new run. If a stall period of less than 15 s was observed between two runs in the same direction, the entire movement was considered as one directional run. The velocities of the directional runs were calculated following the assignment of coordinates to the beginning and end of each directional run (Fig. 3a, left). Each directional run was assigned a “weight” equal to its duration. Calculations of the average velocity and the velocity distribution took the weight of each run into account, with the average being calculated using equation (1) and the SEM using equation (2). The velocity distribution histogram was built using equation (3):


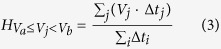


where H is the height of a column at a specific velocity range (which corresponds to the normalized fraction of the duration of runs in the specific velocity range), V_a_ and V_b_ are the borders of the velocity range, V_j_ represents velocities within the range V_a_ < V_j_ < V_b_, Δt_j_ is the corresponding duration of the runs, and t_i_ is the time for all runs. Equations 2–3 were developed for this study.

MSD analysis of Cin8 movement was obtained by tracking the location of a single Cin8 molecule using the ImageJ Spot Tracker plug-in. MSD values were only recorded for runs longer than 630 nm, with a stall time of less than 15 s. Two movements of the same molecule more than 15 s apart were considered as two separate movements. The results of MSD analysis were plotted versus time intervals on a log-log scale and fitted to linear equation^46^,^47^ using OriginPro software.

The statistical significance of differences was determined using a two-tail Student’s t-test.

## Additional Information

**How to cite this article**: Shapira, O. and Gheber, L. Motile properties of the bi-directional kinesin-5 Cin8 are affected by phosphorylation in its motor domain. *Sci. Rep*. **6**, 25597; doi: 10.1038/srep25597 (2016).

## Figures and Tables

**Table 1:** Net velocity of full Cin8 runs.

Cin8 variant	Buffer (IS)	Numberof movingmolecules	Average netvelocity of fullruns (nm/s)	% of full runswith negativenet velocity	% of full runswith zero netvelocity
ATP					
Cin8	MB-30 (0.172)	61	1 ± 2 **^(a)^	47	5
Cin8-3A		59	0 ± 1 **^(a)^	47	6
Cin8-3D		73	−10 ± 4	59	3
Cin8	MB-80 (0.222)	124	−29 ± 6 *^(b)^	64	8
Cin8-3A		92	−9 ± 4	65	4
Cin8-3D		N.A.			
ADP					
Cin8	MB-30 (0.172)	48	−1 ± 1	54	7
Cin8-3A		54	−1 ± 1	46	16
Cin8-3D		40	−2 ± 4	46	0
Cin8	MB-80 (0.222)	42	−7 ± 8	43	11
Cin8-3A		56	−4 ± 7	50	4
Cin8-3D		N.A.			

Weighted average net velocity of full runs of Cin8 variants in whole cell extract with 4 mM ATP or ADP. Velocity was measured for the whole deration of the MT-motor interaction, up to a maximum of 90 s for molecules that did not detach from the MT. The weighted averages ± SEM were calculated taking into account the duration of each interaction using equations 1 and 2. Negative velocity represents movement to the minus-end. (**a**) t-test of the values obtained as compared to that of Cin8-3D at the same IS conditions. (**b**) t-test of the values obtained as compared to that of the same variant at low IS conditions. *P < 0.001; **P < 0.03.

**Table 2:** Interaction of phospho-variants of Cin8 with MTs.

Cin8variant	Buffer (IS)	Number ofmoving molecules	% of moving moleculeswith interaction timeslonger than 90 s
Extract			
Cin8	MB-30 (0.172)	61	75
Cin8-3A		59	72
Cin8-3D		70	23
Cin8	MB-80 (0.222)	126	21
Cin8-3A		92	36
Cin8-3D		N.A.	
Pure			
Cin8	MB-45 (0.187)	85	71
Cin8-3A		69	61
Cin8-3D		232	26
Cin8	MB-90 (0.232)	177	53
Cin8-3A		103	42
Cin8-3D		N.A.	
Cin8	MB-135 (0.277)	118	14
Cin8-3A		128	10
Cin8-3D		N.A.	

The percentages of moving molecules with interaction times longer than 90 s were calculated from kymographs of the three phospho-variants. The percentage of moving molecules that did not detach from the MT throughout the duration of the measurement out of the total number of moving molecules is provided. Sample type, the variant considered and buffer and IS conditions are indicated. Measurements were performed on polarity-marked MTs. For the Cin8-3D variant, no movements were detected in the MB-90 and MB-135 buffers for purified samples and in the MB-80 buffer for the crude extract.

**Table 3:** Yeast strains and plasmids used in this study.

Strain#	Genotype	Description
Yeast strains		
LGY 2055	*MATa, ura3, leu2, his3, lys2, ade2, cin8*Δ*::URA3, leu2::CIN8-3GFP-LEU2*	Cin8-WT whole extract
LGY 2058	*MATa, ura3, leu2, his3, lys2, ade2, cin8*Δ*::URA3, leu2::cin8-3A-3GFP-LEU2*	Cin8-3A whole extract
LGY 2575	*MATa, ura3, leu2, his3, lys2,cin8*∆*::URA3, leu2::cin8-3D-3GFP-LEU2*	Cin8-3D whole extract
LGY 4054	*MATα, leu2-3,112, reg1-501, ura3-52, pep4-3, prb1-1122, gal1* (*pOS2*)	Cin8-WT overexpression for purification
LGY 4057	*MATα,leu2-3,112,reg-1-501, ura3-52, pep4-3, prb1-1122,gal1* (*pOS6*)	Cin8-3A overexpression for purification
LGY 4066	*MATα,leu2-3, 112, reg-1-501,ura3-52,pep4-3, prb1-1122,gal1* (*pOS5*)	Cin8- 3D overexpression for purification
**Plasmids**		
** Plasmid#**	**Description**	
pOS2	*2μ, LEU2, P*_*GAL1*_*-CIN8-3GFP-6His*	
pOS5	*2μ,LEU2,P*_*GAL1*_*-cin8-3D-3GFP-6His*	
pOS6	*2μ,LEU2,P*_*GAL1*_*-cin8-3A-3GFP-6His*	
